# Implementing patient derived organoids in functional precision medicine for patients with advanced colorectal cancer

**DOI:** 10.1186/s13046-023-02853-4

**Published:** 2023-10-25

**Authors:** Jérôme Cartry, Sabrina Bedja, Alice Boilève, Jacques R. R. Mathieu, Emilie Gontran, Maxime Annereau, Bastien Job, Ali Mouawia, Pierre Mathias, Thierry De Baère, Antoine Italiano, Benjamin Besse, Isabelle Sourrouille, Maximiliano Gelli, Mohamed-Amine Bani, Peggy Dartigues, Antoine Hollebecque, Cristina Smolenschi, Michel Ducreux, David Malka, Fanny Jaulin

**Affiliations:** 1 Inserm U-1279, Gustave Roussy, Université Paris-Saclay, Villejuif, F-94805 France; 2 Département de Pharmacie Clinique, Gustave Roussy, 94805 Villejuif, France; 3 Inserm US23, Plateforme de Bioinformatique, Gustave Roussy, 94805 Villejuif, France; 4 Département de Radiologie Interventionnelle, Gustave Roussy, 94805 Villejuif, France; 5 UFR Médecine, Université Paris-Saclay, 94270 Le Kremlin-Bicêtre, France; 6 Département d’Innovation Thérapeutique et d’Essais Précoces, Gustave Roussy, Villejuif, 94805 France; 7 Gustave Roussy, Unité de Médecine de Précision, 94805 Villejuif, France; 8 Département de Médecine Oncologique, Gustave Roussy, Université Paris-Saclay, 94805 Villejuif, France; 9 Département de Chirurgie Viscérale, Gustave Roussy, 94805 Villejuif, France; 10 Département de Pathologie, Gustave Roussy, 94805 Villejuif, France; 11 Département d’Oncologie Médicale, Institut Mutualiste Montsouris, Paris, France; 12 Département de Recherche, Gustave Roussy, 94800 Villejuif, France

**Keywords:** Organoids, Precision medicine, Colorectal cancer, Chemogram

## Abstract

**Background:**

Patient Derived Organoids (PDOs) emerged as the best technology to develop ex vivo tumor avatars. Whether drug testing on PDOs to identify efficient therapies will bring clinical utility by improving patient survival remains unclear. To test this hypothesis in the frame of clinical trials, PDO technology faces three main challenges to be implemented in routine clinical practices: i) generating PDOs with a limited amount of tumor material; ii) testing a wide panel of anti-cancer drugs; and iii) obtaining results within a time frame compatible with patient disease management. We aimed to address these challenges in a prospective study in patients with colorectal cancer (CRC).

**Methods:**

Fresh surgical or core needle biopsies were obtained from patients with CRC. PDOs were established and challenged with a panel of 25 FDA-approved anti-cancer drugs (chemotherapies and targeted therapies) to establish a scoring method (‘chemogram’) identifying in vitro responders. The results were analyzed at the scale of the cohort and individual patients when the follow-up data were available.

**Results:**

A total of 25 PDOs were successfully established, harboring 94% concordance with the genomic profile of the tumor they were derived from. The take-on rate for PDOs derived from core needle biopsies was 61.5%. A chemogram was obtained with a 6-week median turnaround time (range, 4–10 weeks). At least one hit (mean 6.16) was identified for 92% of the PDOs. The number of hits was inversely correlated to disease metastatic dissemination and the number of lines of treatment the patient received. The chemograms were compared to clinical data obtained from 8 patients and proved to be predictive of their response with 75% sensitivity and specificity.

**Conclusions:**

We show that PDO-based drug tests can be achieved in the frame of routine clinical practice. The chemogram could provide clinicians with a decision-making tool to tailor patient treatment. Thus, PDO-based functional precision oncology should now be tested in interventional trials assessing its clinical utility for patients who do not harbor activable genomic alterations or have developed resistance to standard of care treatments.

**Supplementary Information:**

The online version contains supplementary material available at 10.1186/s13046-023-02853-4.

## Background

Over the past decades, the management of patients with metastatic colorectal cancer (mCRC) has only slightly improved since the approval of chemotherapy-based regimens combining 5-fluorouracil, oxaliplatin, irinotecan, epidermal growth factor receptor inhibitors, and antiangiogenic agents [1]. Most patients benefits for these therapies but disease control remains timely limited due to the emergence of drug resistance leaving the patients with few therapeutic options. Moreover, the difference among patients’ response to approved-standard CRC chemotherapies exemplifies inter-patient tumor heterogeneity. This hallmark of cancer requires adapting the therapeutic strategy for each specific patient.

Molecular Personalized Medicine (MPM) approaches try to overcome inter-patient’s tumor heterogeneity by detecting tumor-specific characteristic using protein-, RNA- and genome-based approaches [2]. However, pan-solid tumor clinical trials have shown that MPM benefits only to a small subset (10–15%) of the patients [3–6]. MPM is even more limited at predicting the response to chemotherapies whose effectiveness does not correlate to a single molecular alteration. For these reasons, it is crucial to develop alternative strategies to predict treatment response for each patient. In this regards, Functional Precision Medicine (FPM) is emerging as a promising technology to fulfil this gap. FPM is based upon an *ex-vivo* drug-test in which living tumor cells from a specific patient are exposed to a panel of anti-cancer drugs. This assay aims at identifying the drug sensitivity and resistance profile of each individual tumor to orient patient treatment efficiently [7]. Recently, Malani et al*.* and Kornauth et al*.* showed excellent results in FPM-based strategies for patient with hematological malignancies [8, 9]. These studies pave the way toward the implementation of FPM in these pathologies which provide a fast and easy access to large quantities of tumor cells. Nevertheless, applying FPM to patients with solid tumors has been challenged by the lack of adequate technology to amplify tumor cells ex vivo rapidly and faithfully. Cell lines are poor cancer surrogates, and patient-derived xenograft models are too costly and time-consuming for therapeutic strategies [10]. Recent studies suggest that patient derived organoids (PDO) could hold the promise of FPM for solid cancers. PDO are tridimensional, multicellular structures, expanded in vitro, which retain morphological, histological and genetic properties of their tumor of origin [11]. Furthermore, they are stable over time, self-organized and self-renewing structures. PDO collections have been established for a large variety of tumors, mainly carcinomas, including breast [12], colorectal [13, 14], pancreatic [15, 16] and ovarian [17, 18] cancers. PDOs are currently emerging as powerful pre-clinical models and are suitable for drug screening strategies [11]. In a pooled analysis, the specificity and sensitivity of PDO-based predictive scores exceeded 70% [19]. However, these studies included a very small number of patients and were performed in very favourable settings; including large tumor specimens, no time constraints, single drug tests and no predefined cut-off of sensitivity scores, raising concerns regarding the feasibility to implement PDO technology in routine practice [19]. Moreover, while these studies suggest the clinical validity of PDOs to identify drug sensitivities, they did not assess clinical significance, i.e. the benefit for patients.

To implement PDO-based drug tests in the clinical path of patients with solid tumors requires solving three challenges: 1) establishing PDOs from a limited amount of tumor material obtained by needle biopsy; 2) testing a large panel of anti-cancer drugs (or drug combinations) to increase the probability to identify efficient therapies; and 3) providing the results to clinicians in a timely manner to avoid any interruption in patient therapeutic management. Here, we report a feasibility study of PDO-based FPM in patients with CRC. This includes robust methodology for generating PDOs from core needle biopsies, a test based on a 25-drug panel (named chemogram) and a scoring strategy whose execution is compatible with the constraints of clinical practice.

## Experimental procedures

### Human primary specimens

The human study protocols followed all relevant ethical regulations in accordance with the Declaration of Helsinki principles. Fresh tumor tissues were obtained after surgery or core needle biopsy within two institutional review board-approved, ethics committee-approved precision medicine studies (STING, NCT0493252; MATCH-R, NCT02517892). All patients signed informed consent.

### Tumor digestion

Tumors were minced into pieces and carefully homogenized mechanically. The tumor mix was split into 3 samples of 150 mg carefully weighted with a high precision scale. The first sample was processed according to manufacturer protocol (Tumor Dissociation Kit (human), GentleMACS Disssociator, Milteny Biotec). The second and third samples were incubated in basal medium for 1 h while shaking with Collagenase A (type IV from Clostridium histolyticum, Sigma-Aldrich) for sample 2 or with 50 µg/ml liberase TH (Roche) for sample 3. After digestion, the three samples were processed the same way to avoid any technical bias. Briefly, digestion was stopped with fetal bovine serum (FBS) and cells washed and spun three times. Viability assay was performed in triplicate with 20 μl of the cell solution according to CellTiter-Glo manufacturer protocol (Promega). Number of viable cells was assessed using Kova Glasstic Slide (Fischer Scientific) after trypan blue staining. Two different tumor samples originating from two different patients were used for the test. Three counts were performed by two different researchers. Viability and number of viable cells were normalized to 1 mg of tumor.

### Tumor cell isolation and organoid culture

Tumors were minced into pieces and incubated in basal medium with 50 µg/ml liberase TH (Roche) and 10 µM Y-27632 (Selleckchem) for 1 h while shaking. After incubation, mechanical forces (pipetting) were applied to improve the digestion process. FBS (10%) medium was added, and the mixture filtered through a 100 µm cell strainer. Cells were spun at 350 g for 5 min and the pellet resuspended in red blood cell lysis solution (Milteny Biotec) according to manufacturer procedure. The cell solution was spun at 350 g for 5 min (twice) and the pellet resuspended in basement membrane extract (BME, Matrigel, Corning) and plated. Once embedded in BME, cells were incubated at 37 °C in culture media modified from Fujii et al. [20] and supplemented by Intesticult™ Organoid Growth Medium (Stemcell™ Technologies; 06010), renewed 3 times a week. PDOs were passaged every 7 to 14 days. PDOs were incubated for 5 to 20 min at 37 °C in TrypLE 1X (Thermo Fischer Scientific) and dissociated into single cells and small clusters (< 10 cells) by applying mechanical force (pipetting) every 5 min. After incubation, 10% FBS medium was added, and the cells were spun at 350 g for 5 min (twice). The pellet was resuspended in BME at appropriate ratio (500 cells/μl of BME) and plated. After BME polymerization, PDO culture media containing 10 µM Y-27632 was added and the culture plates incubated at 37 °C.

The 25 PDOs were cryopreserved in FBS containing 10% DMSO (Sigma- Aldrich) and all of them have been tested successfully for culture after thawing. PDOs nomenclature: CGR for Colon Gustave Roussy. The number assigned to each PDO such as 0001, 0002, etc. corresponds to the order in which the surgical or biopsy specimens were processed in the laboratory.

### Histology procedures

PDOs embedded in BME were incubated for 1 h with 4% paraformaldehyde (PFA) at room temperature. After the incubation, the mixture was spun 5 min at 100 g. The PDO pellet was resuspended in PBS and spun 5 min at 100 g. After one more washing step, the PDO pellet was dehydrated with ethanol and embedded in paraffin. Sections were subjected to H&E staining. For detection of CDX2, paraffin sections were processed in a Ventana Benchmark Ultra automated immunostainer instrument for heat-induced antigen retrieval (CC1 Buffer equivalent pH8) for 64 min at 95 °C. Sections were incubated with rabbit monoclonal anti-CDX2 (Roche; #760–4380, clone EPR2764Y, pre-diluted) for 32 min at 36 °C. The signal was revealed with UltraView universal DAB detection kit (Roche #760–500). Finally, the sections were counterstained with hematoxylin and bluing reagent. For detection of CK20, paraffin sections were processed in a Bond Leica automated immunostainer instrument for heat-induced antigen retrieval (ER2 corresponding EDTA buffer pH9). Slides were incubated with CK20 antibody (rabbit monoclonal: LS Bio # LS-C210303, clone SP33, 1:25) for 60 min at room temperature and detected by Bond Polymer Refine Detection kit. The signal was revealed with DAB and slides were counterstained with hematoxylin.

### Whole-Exome sequencing and molecular alteration analysis

Read sequences were evaluated for their quality using FastQC v0.11.9. FastqScreen v0.15.1 was also used to assess for any DNA contamination by other species. Sequences were trimmed for their lower quality (BaseQ < 20) and Illumina adapter sequences using Fastp v0.23.2 [21]. All QC results were compiled to a user-friendly report using multiqc v1.14. Mapping was performed against the human hg19 genome sequence using bwa mem v0.7.17 [22]. Duplicate reads marking and base quality recalibration were performed using GATK v3.8–1-0 [23]. Germline variant/indel calling was performed with Varscan mpileup2cns v2.4.3 [24], using the default parameters. Called variants were then filtered in using bcftools v1.9 according to the following criteria: 1) AD >  = 10; 2) Freq > 5; 3) Func_refGene =  = ‘exonic,splicing’; 4) gnomAD_exome < 1e-03; 5) ExonicFunc_refGene ! = ‘synonymous_SNV’; 6) ExonicFunc_refGene ! = ‘unknown’. Filtered variants quality was assessed using bcftools stats.

The original tumor molecular alterations were extracted from the medical record of the patients and detailed in Fig. 2D and Supplementary Table 2.

### Copy number alteration analysis

Identification of copy-number anomalies were performed using EaCoN v0.3.6 [25] under R v3.6.2, for all the steps described hereafter. All organoid profiles were compared to a sex-matched in-house reference patient profile (MRA1012_N for females, MRA1144_N for males). GATK base-recalibrated BAM files were internally transformed to the mpileup format using Rsamtools v2.8.0 [26] ignoring replicates and secondary alignments. To generate the log2ratio data, the test and reference mpileup profiles were binned to windows of 50 nt in median (depending on the capture BED information), and bins with a total depth < 20 were discarded. Using a pre-generated track of GC% content in bins, those with a value < 20% or > 80% were flagged as outliers. The log2ratio (L2R) of test / reference depths was computed for each bin, and linearly imputed for GC% outliers. The L2R was then normalized for GC% using a lowess regression. To generate the BAF data, any non-reference sequences in the mpileups were identified and their depth quantified. SNP variants supported by less than 3 reads and/or for which the total depth was below 20 were discarded. To filter for noisy, low frequency variants, all SNP variants in the test sample with a reference frequency below 33% were discarded. The bivariate (L2R and BAF) data were then segmented, evaluated for their allele-specific and absolute copy-number, as well as ploidy and tumor cellularity, using ASCAT v2.5.2 [27]. CGR0009 WES data could not be exploited for the CNA analysis.

### Drug tests (chemograms)

All the drugs tested were purchased at Clinisciences, except oxaliplatin and carboplatin, which were provided by Gustave Roussy hospital pharmacy. The stock concentration was 10 mM. The solvent was DMSO apart from oxaliplatin, carboplatin and trifluridine-tipiracil for which PBS-tween20 (0.3%) was used. 96-wells drug source plates were prepared with a D-300e digital dispenser (Tecan). The drug concentration in the source plates was 10 times higher than the desired final concentration and the drugs were dissolved in 100 μl of PDO culture media. PDOs tested were incubated for 20 min at 37 °C in TrypLE 1X and dissociated to single cells by applying mechanical force (pipetting) every 5 min. Cells were counted using Kova Glasstic Slide, embedded in BME in a 250 cells /μl ratio and plated (3 μl per well) in the 60 center wells of a 96 wells plates with a pipetting robot (Assist Plus, Integra). The BME domes were overlaid with 125 μl of PDO culture media with a pipetting robot (Viaflo, Integra). Two days post seeding, media was removed and replaced by 112.5 μl of fresh PDO culture media. 12.5 μl of the drug source plate was added using a pipetting robot (Viaflo, Integra). Media and drugs are renewed at day 6. At day 8, media was removed and 50 μl of Cell Titer Glo 3.0 (Promega) diluted by 2 in basal culture media was added to each well. Culture plates were agitated for 5 min on an orbital shaker and luminescence was recorded after 20 min of incubation at room temperature.

The intracellular LDH level was measured using the LDH-Glo assay (Promega) according to manufacturer protocol after PDO lysis with 100 µl of PBS/Triton 1% per well. Lysate was diluted by 600 in PBS. 50 µl of the diluted lysate were incubated with 50 µl of LDH-Glo reagent for 1 h.

Readings of bioluminescence were obtained using Biotek Synergy H1 plate reader. Each condition was tested in triplicate (3 wells). Control wells were containing solvent but no drug.

### PDO imaging assay

The microcavity 96 wells Eplasia plate system (Corning) was used according to manufacturer protocol after PDO dissociation into single cells using TrypLe 1X. Drug were added 2 days after cell loading using a D-300e digital dispenser (Tecan). PDOs were imaged with a LionHeart FX (BioTeK Agilent) and segmented with Gen5 software.

### Chemogram scoring system

For each condition, the average value and the standard error of the three wells (ATP-bioluminescence signal) were calculated. The average value of each condition was normalized to the average value of the control wells providing the percentage of relative viability. When the standard error was over 12% the data was excluded from the analysis or the test redone. The Area Under the Curve (AUC) for each of the 25 PDO and each of the 25 drugs was calculated using excel analysis. For each drug, the AUC of the average response of the 25 PDOs was divided by the AUC of the PDO of interest. The result was named AUC score. The direct response of the PDO to each drug to the 3 concentrations tested was also calculated and called the sensitivity score. This score was defined by calculating the ratio between the area over the curve and the total area. The AUC score was summed to the sensitivity score to give rise to the final score used in this study.

## Results

### Operate PDO establishment in clinical setting

**Fig. 1:**
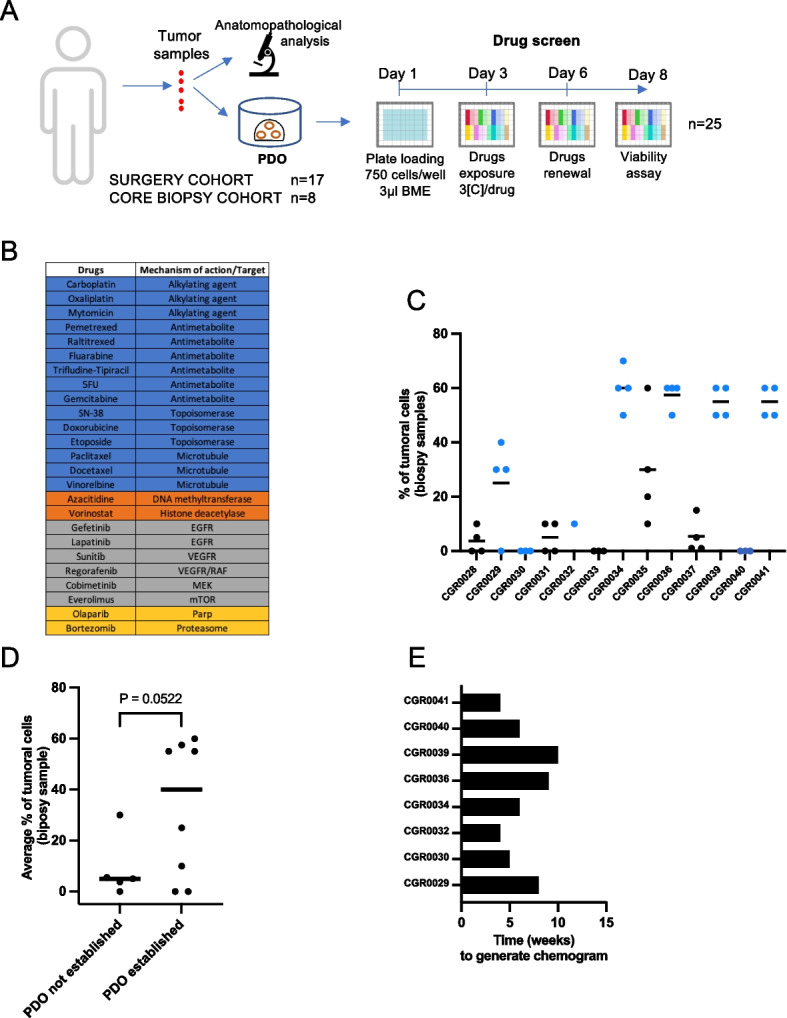
Study design and PDOs derivation. **A** Overview of the procedure. **B** Mechanism of action or target of the drugs used in the study (blue: chemotherapies, orange: epigenetic drugs, grey: kinase inhibitors, yellow: others). **C** Percentage of tumor cells retrieved from core needle biopsy samples. During each biopsy procedure, several tumor samples were taken. Amongst all samples collected, 1 (e.g., CGR0030) to 4 (e.g., CGR0029) of them were assessed for cellularity. Each dot represents one core needle biopsy sample, the dots are blue when the corresponding PDO line has been successfully derived. **D** Average percentage of tumoral cells in the biopsy samples according to PDO establishment. **E** Time (in weeks) lasting from biopsy to chemogram results. Data are presented as each value and mean in **C** and **D**. Significance is determined with unpaired two-tailed t-test with Welch’s correction in **D**

Our primary objective was to develop a protocol to generate PDOs with the smallest amount of tumor material and the fastest expansion rate to timely screen 25 approved anti-cancer drugs. The demographic and clinical data of CRC patients in our study are presented in Table 1. Most patients had metastatic disease (84%) and had received at least one prior treatment line (76%). PDOs were derived either from large surgery specimens (*n = *10), small needle-like specimens obtained from larger fragments (*n* = 7) or bona-fide core needle biopsy samples (*n* = 8) (Supplementary Table 1). The study design and the drug panel are presented in Figs. 1A and B. Importantly, fresh tumor material was immediately collected and processed within 1 h. For the core needle biopsy procedure, several samples were collected: two were dedicated to PDO derivation while the others were treated for histological assessment and tumor cell fraction evaluation. The large tumor specimens recovered from surgeries were used to optimize PDO establishment and amplification. To favor tumor cell recovery and viability, three different digestion protocols were evaluated: tumor dissociation kit (Milteny Biotec), collagenase, and Liberase TH. After digestion of two different tumor specimens to generate single cells, the number of viable cells was evaluated using two independent read-outs (ATP-bioluminescence and cell count). Among the three digestion protocols, the liberase TH released the highest number of viable cells (≈ 10000 cells/mg of tumor, Supplementary Fig. 1A). We therefore adopted this enzyme cocktail for the rest of the study and were able to generate between 300 000 and 400 000 cells to perform the chemogram after a few weeks of amplification. The percentage of tumor cells quantified by pathologists in the core biopsy samples was highly variable among patients (0 to 70%, Fig. 1C). Although distinct biopsy specimens were used for histology assessment and PDO establishment, tumor sample cellularity tended to be associated with the success in PDO generation (Fig. 1D). Ultimately, the average PDO take-on rate from surgery specimens was 63% and 61.5% when derived from needle biopsies. The time of the entire procedure from the date of a core needle biopsy to the end of the chemogram ranged between 4 and 10 weeks depending on the patient with a median of 6 weeks (Fig. 1E).

**Table 1:** Patients characteristics

**Demography**	
Median age (years) (range)	60 (50–72)
Female	16 (64%)
**Disease stage**	
I	2 (8%)
III	1 (4%)
III	1 (4%)
IV	21 (84%)
**Tumor sample**	
Surgery	17 (68%)
Core Needle Biopsy	8 (32%)
**Tumor sample source**	
Primary Tumor	8 (32%)
Colon	5 (20%)
Rectum	3 (12%)
Metastasis	17 (68%)
Liver	15 (60%)
Peritoneum	1 (4%)
Ovary	1 (4%)
**Prior treatment lines**	
0	6 (24%)
1	8 (32%)
2	7 (28%)
3 +	4 (16%)
**Prior drug regimens received**	
FOLFOX	12 (48%)
FOLFIRI	8 (32%)
FOLFIRINOX	8 (32%)
Anti-EGFR	11 (44%)
Anti-VEGFR	9 (36%)
Regorafenib	1 (4%)
Trifluridin/Tipiracil	2 (8%)

### Histological and molecular analysis of PDOs

**Fig. 2:**
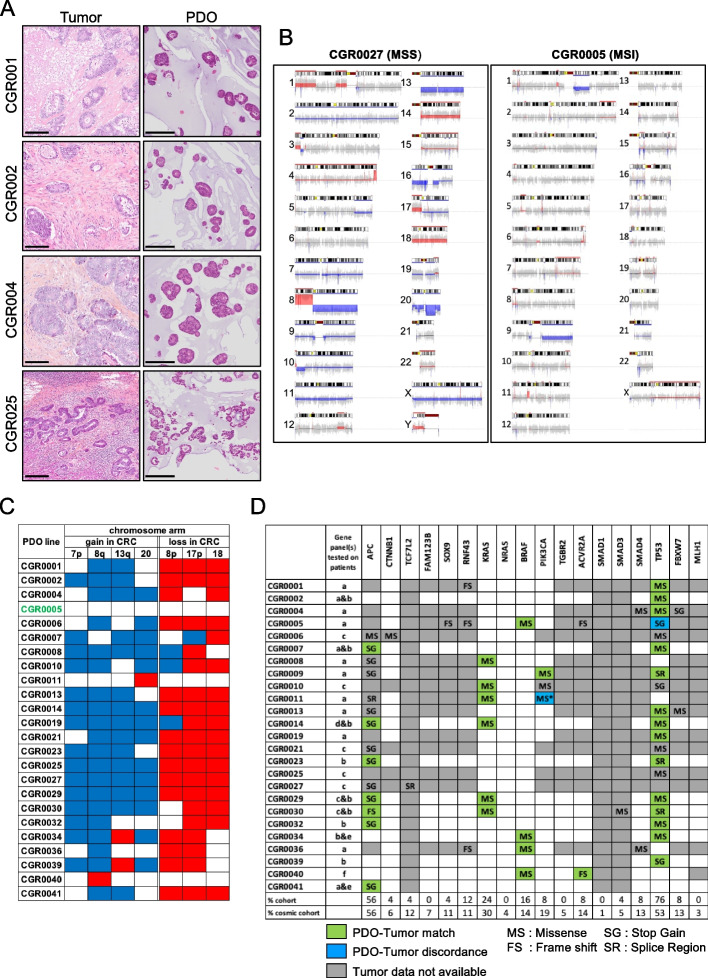
Histological and molecular analysis of PDOs. **A** H&E (hematoxylin/eosin) staining of tumor and matching PDO. Scale Bar, 250 μm. **B** CNA representation of an MSS PDO (left panel) and an MSI PDO (right panel). Blue and Red indicate gain and loss regions respectively. **C** CNA of the 25 PDO lines for 7 chromosome arms frequently altered in CRC carcinogenesis. **D** Oncogenic somatic mutations of the PDOs for 18 genes and comparison with available molecular alterations of matching tumors. The percentage of each mutation in the PDO cohort is compared to the percenatge of mutations observed in the cosmic cohort. MS*: The missense PIK3CA mutation is found in the tumor tissue but not in the matching CGR0011 PDO. The gene panels tested on tumor tissues are detailed in Supplementary Table 2

We next assessed PDO histology and molecular profiles. The comparison between PDOs and original tumors histology revealed remarkable morphological similarities (Fig. 2A for representative examples). The histological grading, the differentiation pattern, and the structure of the PDO were consistent between the PDOs and the patient tumors. The colon origin of the PDOs was confirmed with CDX2 and CK20 staining (Supplementary Fig. 1B). Subsequently, we performed whole exome sequencing (WES) for in depth PDO profiling. First, we analyzed PDO copy number alterations (CNAs). In microsatellite stable CRC (MSS, see CGR0027 for a representative example), recurrent CNA regions with gains were frequent in chromosomes 7p, 8q, 17q and 20 whereas chromosomes 8p, 17p and 18 cumulate losses [28] (Fig. 2B, left panel). In contrast, the PDO CGR0005 derived from microsatellite instability (MSI) CRC harbored minimal CNAs (Fig. 2B, right panel). We summed the CNA analyses for all PDOs from our cohort and observed a robust correlation with the expected gain and loss profiles previously published in MSS CRCs, and genomic stability characteristic of MSI tumors (Fig. 2C). Second, we compared the genomic alterations between patient tumors and their matching PDOs. We compared WES data with available patients’ genomic data for 18 oncogenic drivers (Fig. 2D and Supplementary Table 2). We were able to analyze 35 mutations between PDOs and matching tumors and found a 94% (33/35) concordance. We identified two mismatches, a lack of PIK3CA mutation in the CGR0011 PDO while PDO CGR0005 exhibits a TP53 mutation that was not found in the original tumor. Interestingly, CGR0005 tumor harbors microsatellite instability, in line with the PDO having the highest total number of mutations referenced in the Catalogue of Somatic Mutations in Cancer (COSMIC) [29] (528 versus 175.4 in average, Supplementary Fig. 1C). In accordance with COSMIC database, we identified APC and BRAF mutations in respectively 14 (56%) and 4 (16%) of the PDOs (Fig. 2D, last row). We noticed that our PDO cohort was enriched in TP53 mutations (76% versus 53%) and slightly under-represented for KRAS mutations (24% versus 30%). Overall, we show that the PDOs are faithful avatar of their original tumor. They exhibit a spectrum of mutations consistent with the main alteration of CRC despite slight differences possibly due to recruitment bias such as enrichment in liver metastasis.

### Determining relevant drug concentrations

**Fig. 3:**
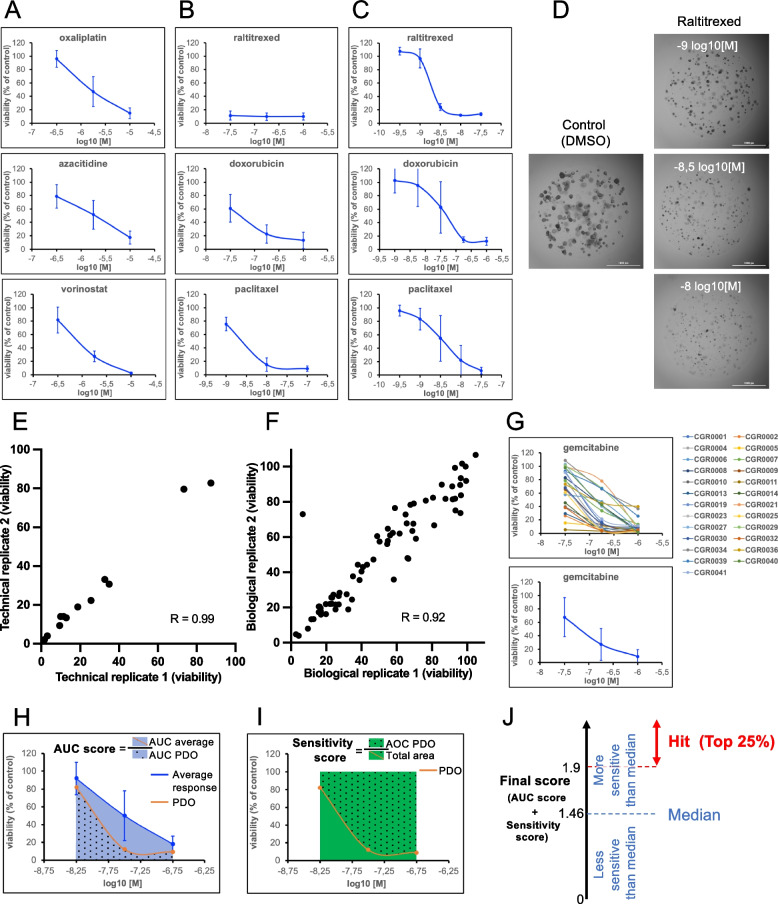
Chemogram Calibration. **A**-**B** Drug concentrations first characterization. Average response of 8 PDOs. **C** Drug concentrations second characterization. Average response of 8 PDOs. **D** Bright-Field imaging at day 8 of CGR0002 PDO exposed to 3 concentrations of Raltitrexed. **E** Drug test technical reproducibility. **F** Drug test biological reproducibility. **G** Relative viability (%) of the 25 PDO lines tested with gemcitabine at 3 concentrations (top panel). Bottom panel shows the average response of the 25 PDO lines to gemcitabine. **H**-**J** Scoring system. **H** AUC score: for each drug the average response of the 25 PDOs to an individual drug is determined (blue line) and allows for average AUC calculation (blue area ± dots). This AUC is divided by the AUC of the response of an individual PDO line (orange line and blue area with dots). **I** Sensitivity score: ratio between the area over the curve (green with dots) and the total area (green ± dots). **J** The AUC score was summed to the sensitivity score to give rise to the final score used in this study. Hit determination according to the median score of the entire dataset (25 PDOs tested with 25 drugs). Data are presented as the mean ± SD in **A**, **B** and **C** (*n* = 8 PDOs), as the mean ± SD in **G** (bottom panel; *n* = 25 PDOs), as the mean of triplicates in **E** (CGR0025 screened twice on the same run with 4 drugs at 3 concentrations (12 dots)), as the mean of triplicates in **F** (CGR0025 screened on two different runs with a 2-week interval for 25 drugs at 3 concentrations (75 dots)). Correlation is determined with the Pearson correlation coefficient (R) in **E** and **F**

We designed a library of 25 drugs approved by the FDA for at least one cancerous indication. The panel includes chemotherapeutics (*n* = 15), kinase inhibitors (*n* = 6) and epigenetic drugs (*n* = 2) to cover a large set of action mechanisms (Fig. 1B). PDOs were treated as follows: after digestion, PDO-derived single cells were embedded in BME and seeded in 96-well plates at 750 cells/well. After 48 h, PDOs were exposed to the drugs at 3 different concentrations for 5 days (renewed at day 3). The activity of the drugs against the PDOs was assessed at day 8 (Fig. 1A). We chose the easy-to-use ATP-bioluminescence viability assay to quantify the effect of drugs on PDOs. This test has been the most widely used to highlight the predictive potential of PDOs [19] and we observed and confirmed an excellent correlation (Pearson correlation coefficient > 0.86) between the ATP-signal and the size of the PDOs (Supplementary Fig. 2A-C) or the intracellular LDH level (Supplementary Fig. 2D) [30]. To determine the relevant drug concentrations to test ex vivo, we used two criteria. First, the concentrations must be consistent with physiological or sub-physiological drug concentration observed in patients [31]. Second, the chosen concentrations must cover the average IC50 of each drug. As a pilot experiment, 8 PDOs were challenged using a first set of concentrations for the 25 drugs. Two profiles were observed: the concentrations either surrounded the average IC50 (Fig. 3A) or were inducing a massive killing effect at 2 or 3 concentrations (Fig. 3B). In this case, PDO were rechallenged with 5 different concentrations to capture the average IC50 (Fig. 3C). As a result, three concentrations (high, medium, and low) were determined for each of the 25 drugs (Table 2). As an example, Fig. 3D shows the effects of 3 concentrations of raltitrexed on CGR0002 PDOs embedded in 3 μl BME drops. The assay proved to have both high technical (Fig. 3E and Supplementary Fig. 3A) and biological reproducibility accross different PDO passages (up to 5) and over freezing and thawing procedures (Fig. 3F and Supplementary Fig. 3B-C). We next used these drug concentrations to challenge the entire collection of 25 PDOs and determine the average dose–response of the PDO cohort (Fig. 3G for gemcitabine as an example and Supplementary Fig. 4 for all other drugs). The lower the dose–response curve for a given PDO is compared to the average, the more sensitive this PDO is to the drug (for instance (Fig. 3G), CGR00011 was the most sensitive to gemcitabine and CGR00021 the most resistant). This method determines how each specific PDO responds to a specific drug as compared to the cohort.

**Table 2:** Final drug concentrations, log10 [M]

**Drugs**	**[C] low**	**[C] medium**	**[C] high**
5FU	-7	-6.25	-5.5
Azacitidine	-6.5	-5.75	-5
Bortesomib	-9.25	-8.5	-7.75
Carboplatin	-6.25	-5.5	-4.75
Cobimetinib	-8	-7.25	-6.5
Docetaxel	-9.25	-8.75	-8.25
Doxorubicin	-8	-7.25	-6.5
Etoposide	-6.75	-6	-5.25
Everolimus	-8.5	-7.75	-7
Fludarabine	-8	-7.25	-6.5
Gefetinib	-7.5	-6.75	-6
Gemcitabine	-7.5	-6.75	-6
Lapatinib	-7	-6.25	-5.5
Mitomycin	-8	-7.25	-6.5
Olaparib	-6.5	-5.75	-5
Oxaliplatin	-6.5	-5.75	-5
Paclitaxel	-9	-8.5	-8
Pemetrexed	-8.25	-7.5	-6.75
Raltitrexed	-9	-8.5	-8
Regorafenib	-6.5	-5.75	-5
SN-38	-9	-8.5	-8
Sunitinib	-7	-6.5	-6
Trifluridin-tipiracil	-6.5	-5.75	-5
Vinorelbine	-8.75	-8	-7.25
Vorinostat	-6.75	-6.25	-5.75

### Identifying ex vivo responders

We then established a scoring system to rank drug sensitivity for each PDO. To account for the specific distributions and profiles of each average dose–response curve, we set-up a two-step scoring method. First, we determined, for each drug, the area under the curves (AUC) of the average and specific response of the 25 PDOs (Fig. 3H and Supplementary Fig. 4). We calculated a ratio to evaluate how responsive is a specific PDO as compared to the PDO collection (Fig. 3H). In some cases, the AUC ratio is high, though there is a modest reduction in viability at the chosen physiological concentrations (e.g., lapatinib or gefitinib, Supplementary Fig. 4). To account for this, a second step evaluates a sensitivity score representing the direct response of the PDO to each one of the three drug concentrations (Fig. 3I and methods). The AUC score and the sensitivity score are summed to obtain the final score. The median final score of all the drugs on all the PDOs is 1.46 and the 3^rd^ quartile 1.9. In consequence, we defined as a hit a drug showing a score > 1.9 for a particular PDO indicating a strong reduction of the PDO viability (Fig. 3J).

### Profiling the drug response landscape in CRC PDO collection

**Fig. 4:**
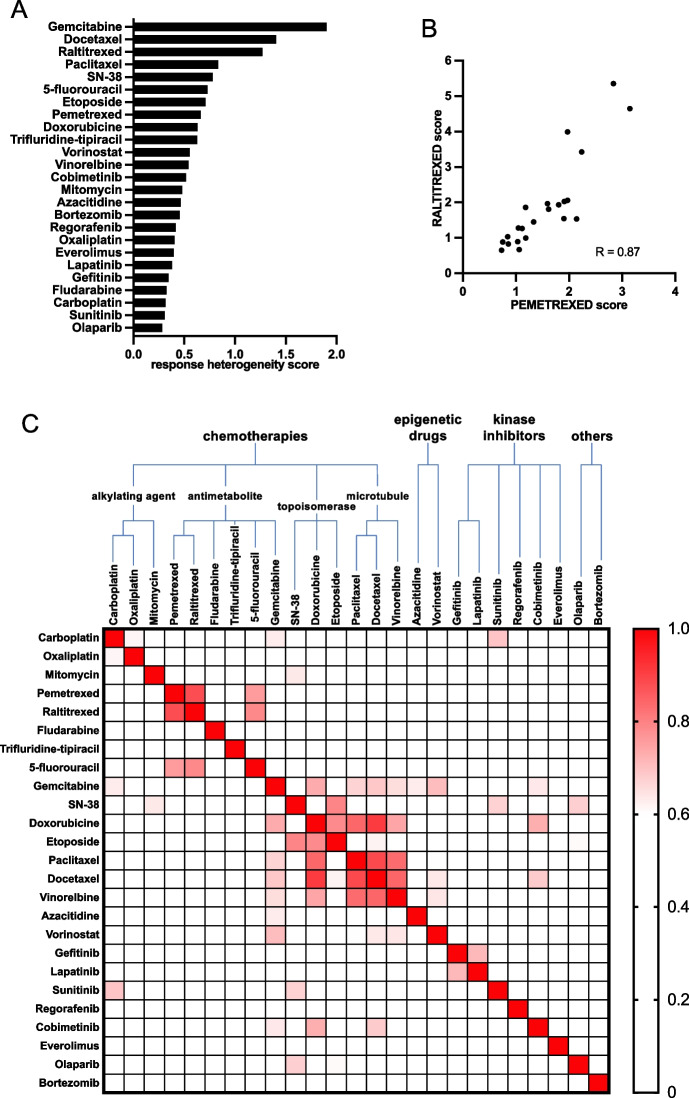
Drug test correlation. **A** Response heterogeneity score (standard deviation of the scores) calculated for the 25 PDOs to each drug. **B** Scatterplot of the 25 PDOs score to both Pemetrexed and Raltitrexed. Each dot represents one PDO. **C** Drug Correlation matrix. The score of the 25 PDO lines to all drugs is analyzed with a Pearson correlation test in **C**. Color coding highlights the correlation based on the Pearson correlation coefficient (R)

Based on the above-mentioned drug concentrations and scoring system, the drug response of the entire PDO cohort was then profiled. The degree of response heterogeneity of the PDOs to the 25 drugs was variable. Some drugs showed very different responses across the PDOs (e.g., gemcitabine, Fig. 4A), others much less (e.g., olaparib). We then looked at the correlation between the response to drugs sharing a similar mechanism of action. As an example, raltitrexed and pemetrexed, two folic acid analogs, harbor highly correlated responses across the PDO collection (R = 0.87, Fig. 4B). This excellent correlation is an important internal quality control, validating our concentrations, assay and scoring method. We then used hierarchical clustering based on Pearson's score to investigate correlation between the 25 drugs of the panel (Fig. 4C). We identified a robust correlation between the 3 microtubule-targeting agents (paclitaxel, docetaxel and vinorelbine) as well as between targeted therapies against EGFR (lapatinib and gefitinib) or the platinum salts (carboplatin and oxaliplatin) (Supplementary Fig. 5A). We also found other correlations > 0.5 that could not be explained by the similar nature of the molecules and could provide interesting area of investigations.

**Fig. 5:**
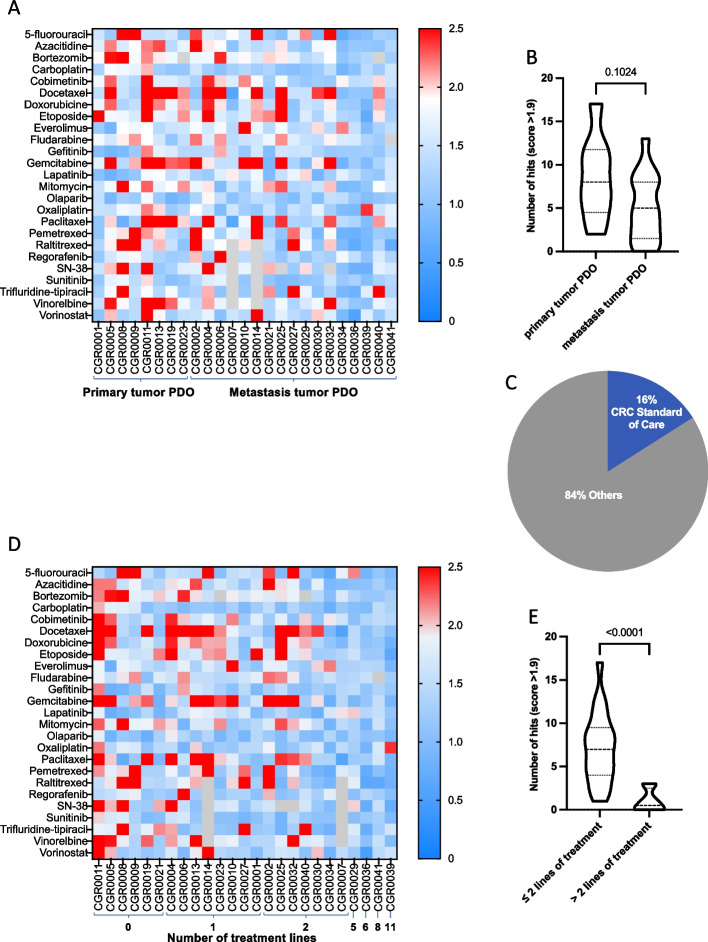
Drug response landscape across the PDOs collection. **A** Heatmap of scores of all 25 drugs tested on the 25 PDOs. Grey square indicates not tested. PDOs were clustered according to origin of the tumor samples (primary or metastasis). **B** Number of hits identified in primary tumor and metastasis derived PDO. **C** Percentage of hits belonging to the CRC therapeutic arsenal versus drugs with authorization in other pathologies. **D** Heatmap of PDO scores to each of the 25 drugs tested. PDOs were clustered according to the number of treatment lines received by the patients pre-surgery/biopsy used to generate the PDO was done. **E** Numbers of hits per PDO according to the number of previous treatment lines. Data are presented as a violin plot with median (dashed line), 1^st^ and 3^rd^ quartiles (dotted lines) in B and E. Significance is determined with unpaired two-tailed t-test with Welch’s correction in **B** and **E**

Finally, we used heatmaps to represent the PDOs response to the drug panel, higher (red) and lower (blue) values, indicating respectively higher and lower sensitivities to drugs (Fig. 5). The average number of hits per PDO was 6.16, ranging from 0 (CGR0036 and CGR0041) to 17 (CGR0011, Fig. 5A). The number of hits tended to be higher for PDOs derived from primary tumor (8.5 in average) than for those generated from metastases (5.1 in average, Fig. 5B). Overall, 84% of the hits were drugs approved in other tumor types but not standard of care (SOC) in CRC (Fig. 5C and Supplementary Fig. 5B). Lastly, we analyzed the drug response as a function of the number of treatment lines the patients were exposed to before biopsy. The heatmap and statistical analyses demonstrated an inverse correlation between the number of hits and the number of treatment lines (Fig. 5D and E).

### Case report: PDO predicts patient response to oxaliplatin

**Fig. 6:**
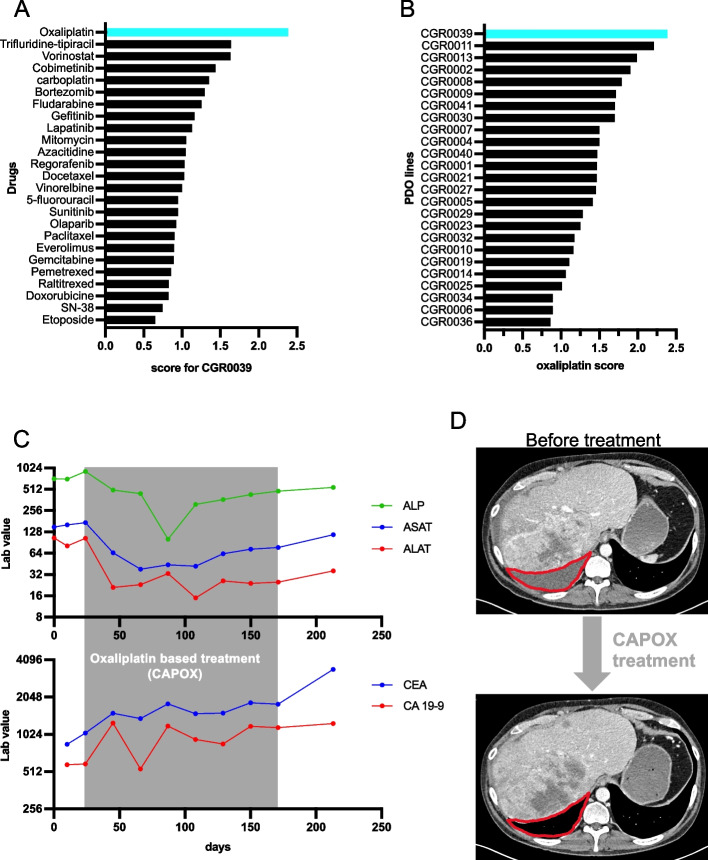
Case report. **A** Score of the 25 drugs tested on CGR0039 PDO. **B** Oxaliplatin score for the 25 PDOs. **C** Lab value of ALP, ASAT, ALAT (liver function) ACE and CA 19–9 (tumor marker) blood levels collected from the biopsy date (day = 0) up to 50 days after the end of the CAPOX treatment. **D** CT-scan before and 2 months after the beginning of the CAPOX treatment. Red line delimits the peritoneal effusion before treatment which is not observed after 2 months of CAPOX treatment

Out of the 25 patients who were included in our study, the case of patient CGR0039 is particularly striking. He was diagnosed with a CRC metastatic to the liver in 2014. Before we generated the PDO from his tumor in 2021, he received 11 lines of treatment including systemic and hepatic arterial chemotherapies. Surprisingly, oxaliplatin was the best hit for this patient and his PDO was the best responder to oxaliplatin among the entire cohort (Fig. 6A and B). After the biopsy used to generate the PDO, this patient was rechallenged with an oxaliplatin-based regimen (capecitabine and oxaliplatin: CAPOX). During his treatment, the patient presented a clear biological benefit with an improvement in liver enzymes (ALP, ASAT and ALAT, Fig. 6C) and stabilization of tumor markers (CEA and CA19.9). Comparing CT-scan before and after oxaliplatin-based regimen showed the disappearance of a large tumor peritoneal effusion, proving a significant clinical benefit (Fig. 6D). Thus, this patient-derived PDO exhibits an unexpected sensitivity to oxaliplatin, in line with the patient response to oxaliplatin-based regimen he received after his biopsy. The patient eventually progressed after 6 months of CAPOX treatment.

### Clinical concordance

**Fig. 7:**
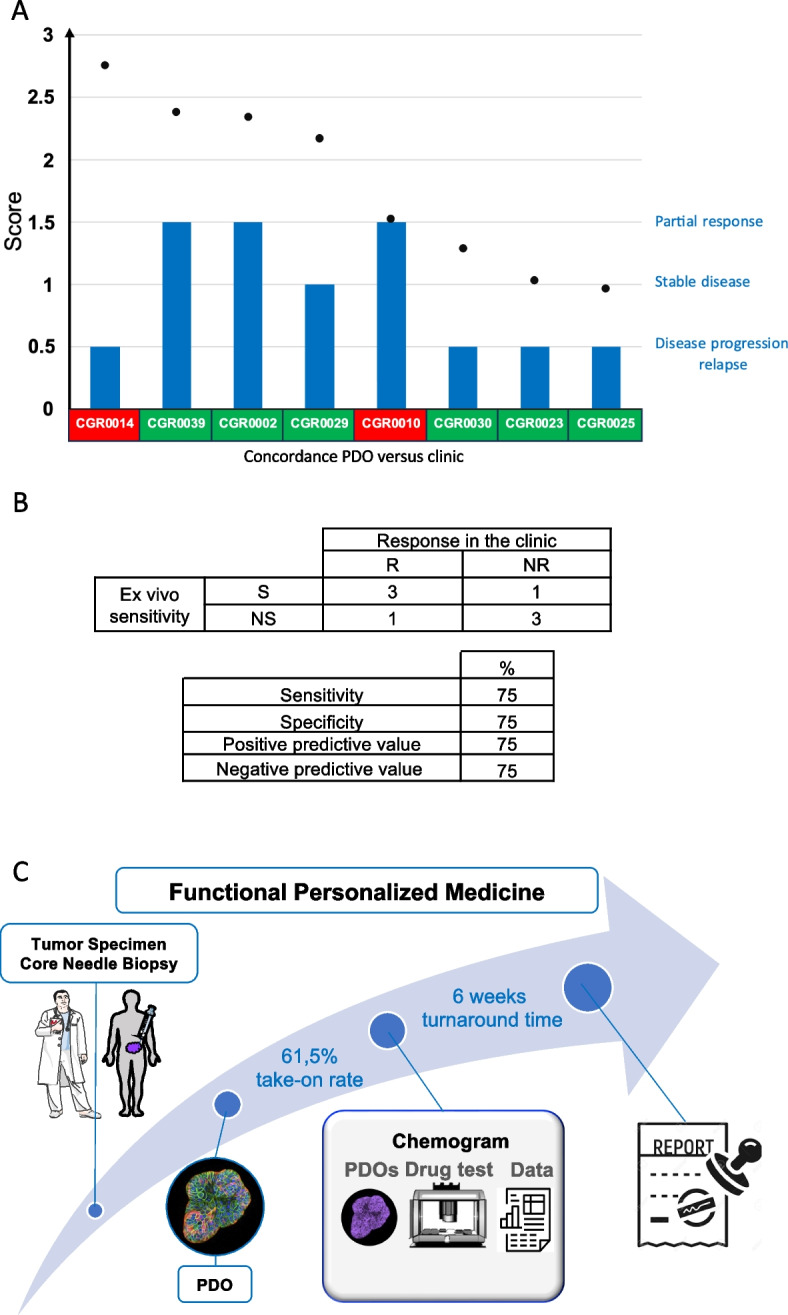
Clinical concordance between PDOs and matching patients. **A** Plotting of drug score (black dots) and clinical response (blue bars) on 8 PDOs with matching clinical data. Green and red rectangles indicate concordance and discrepancy respectively between PDO sensitivity and clinical response. **B** Patient and matching PDO response. S: Sensitive, NS: Non-Sensitive, R: Responder, NR: Non-Responder (upper table). Predictive scores (lower table). **C** Functional Personalized Medicine workflow

In the course of this study, we were able to monitor 13 patients who received treatment after we established their PDOs. Three of them never relapsed after adjuvant therapies, yet we cannot conclude whether they responded to the drug or benefited from a complete ablation of their tumor during surgery. Two patients received treatments that were not included in the chemogram drug panel (e.g., immunotherapies). The 8 remaining patients received a drug included in this panel. Consequently, we could compare the response of the patients and their respective PDOs to the same drug. Matching PDO and patients were sorted based on score and clinical benefit of the treatment (partial response, stable disease or disease progression, Fig. 7A and Supplementary Table 3). For the PDOs that we analyzed, we found 75% sensitivity, specificity, positive predictive value, and negative predictive value in predicting response to drugs in patients (Fig. 7B).

## Discussion

We presented an assay to establish PDOs from core needle biopsies and generate high-quality drug sensitivity data to identify treatment options for patients with advanced CRC (Fig. 7C). We demonstrate that the procedure for PDO generation and amplification fits with deep and fast pharmacological profiling compatible with patient management. As a result, PDOs could be used for precision medicine and increase the chances of identifying effective treatment options.

To the best of our knowledge, two FPM clinical trials on solid tumors have been completed and published: SENSOR [32] and Tumorspheres Colrec [33]. The SENSOR trial was negative with only six patients treated without clinical benefit. The phase 2 Tumorspheres Colrec trial was positive. Nonetheless, among the 34 patients treated based on the results of ex vivo drug tests, only half showed progression-free survival at two months. These two studies highlight that functional precision medicine strategies have still to be improved to benefit cancer patients. Together with previous reports and our own observations, this points to four essential criteria toward clinical implementation [34].

First, the generation of tumor avatars should be delivered for the largest number of patients, regardless of the nature of their tumor (histology, molecular profile). In this regard, the 25 PDO cohort we generated from CRC patients displays molecular heterogeneity. The oncogenic mutations were 94% identical to the ones portrayed in the patients, and consistent with the Cosmic database [29]. The success rate at generating PDO is commonly high when large tumor samples are used as starting material (surgeries or effusions). For instance, Narasimhan et al. reported a PDO take-on rate of 68% in the APOLLO study [35]. Althougt, to be deployed at large and impact patient care, PDO establishment must predominantly start from core needle biopsies. This procedure was used in the SENSOR and Tumorspheres Colrec studies with a PDO take-on rate of respectively 57% and 53.6%. Here, starting with core needle biopsies for 13 patients in the constraint of clinical setting, we report a PDO take-on rate of 61.5%. In addition to the quantity of material, the quality is crucial for PDO derivation success. We found strong heterogeneity in the percentage of tumor cells at the inter- and intra-patient levels that can be explained by random sampling or necrosis induced by previous line(s) of therapy. Correlations between cellularity and take-on rate were also drawn from TUMOROID [36] and SENSOR studies. Mastering the digestion procedure, culture protocols and reducing time from biopsy to the laboratory are key factors to improve the success rate of PDO establishment. Reaching an acceptable take-on rate for clinical implementation can be achieved by multiplying the number of samples retrieved during the biopsy procedure.

As a second criterion, the turnaround time to deliver the results to the clinician is key to inform clinical decisions in a timely manner. This parameter depends on multiple variables including PDO growth rate and the number of drugs tested. This study demonstrates the feasibility of testing as many as 25 drugs in less than 10 weeks (median, 6 weeks). These results are in line with those of SENSOR and Tumorspheres Colrec studies with median times to generate the drug sensitivity data of 10 weeks and 4.8 weeks, respectively. To avoid leaving patients without treatment thoughout the duration of the chemogram, the biopsies could be performed at the onset of the last line of SOC therapy, around 8–16 weeks before receiving the chemogram-guided treatment. In support to this strategy, the SENSOR study used biopsies sampled before and after SOC therapy to show that SOC exposure did not alter the PDO response to the experimental drug tested. Nevertheless, FPM would benefit from shortening assay turnaround time to provide guided treatment as fast as possible to the patients. In this regard, microfluidic technologies, by downscaling the number of cells per condition, are currently emerging as great options to reduce turnaround time [37, 38].

Third, the largest the panel of drugs tested is, the more likely it will identify a therapeutic option (‘hit’) for the patient. Of course, testing a comprehensive number of molecules requires a substantial amount of PDOs and must be balanced with turnaround time and PDO amplification rate. While 8 and 9 drugs or drug combinations (on average) were tested in the SENSOR and Tumorspheres Colrec studies, respectively, our panel included 25 drugs. If we report the number of drugs tested to the number of weeks necessary to obtain the results, our drug screening throughput (25 drugs in 6 weeks) is 6.8 and 2.1 times higher than those in SENSOR and Tumorspheres Colrec studies, respectively. Importantly, guided by the idea of making FPM available for a maximum of patients, we included chemotherapies and targeted therapies that could be afforded by hospitals in academic clinical trials. Also, most of the molecules included in our panel have been tested in metastatic CRC in the frame of clinical trials [39–43]. Despite being negative at the population scale, a large majority of these trials showed a low but significant percentage of clinical response at individual patient scale. This is concordant with our results showing that 84% of hits do not belong to SOC in this indication. This suggests it is relevant to test non-CRC drugs as additional therapeutic options for refractory CRC patients and positions FPM as a strategy to identify therapies that may not have proven benefit at the population scale but still represent relevant therapeutic opportunities at the single patient level.

Setting a robust method to identify patient tumor vulnerabilities based on ex vivo drug tests is an essential fourth criterion. To date, there are two main strategies to identify drug sensitivity ex vivo. On one hand, score and hit identification systems can be built up by comparing FPM results for an individual drug with clinical outcome after treatment with that drug [10, 34]. The scoring system developed can then be used prospectively to orient patient treatment based on FPM results. This approach is suitable for standard of care drugs. For example, in CRC, it is possible to compare the clinical outcome and effect on PDOs of 5-FU, oxaliplatin and irinotecan. However, when the drugs to be tested are not prescribed to the patients in routine, the "PDO-patient" comparison is impossible. On the other hand, to fulfill the fundamental principle of FPM to test a wide range of drugs, we thought, in line with other laboratories [32, 33], to develop an alternative scoring system. This is based on building up a pilot PDO cohort to calculate average responses for each drug at the population scale and being able to to prospectively identify remarkable sensitivities for specific PDOs. Identifying outlier responses defined as “hits” aims at identifying the best therapeutic option for a specific patient across a large panel of anti-cancer drugs that may not be given in their indication.

To do so, we first chose the drug concentration tested to be consistent with the endogenous level that may irrigate the tumor. Second, we adapted the concentrations tested to capture the average IC50 of each drug. Thus, it became possible to observe important drug sensitivity differences between the PDOs. In this way, PDOs can be ranked from best to worse responder for a single drug. Also, our scoring system allows for one PDO to rank the 25 drugs for their in vitro efficiency. In addition to high technical and biological reproducibility (R > 0.92), we also observed a strong correlation between drugs of the same family or sharing the same mechanism of action, validating the chosen concentrations and the scoring system. We believe FPM will benefit from testing related drugs to strengthen the choice of the PDO guided treatment. Interestingly, we observed an inverse correlation between the numbers of hits found and the number of lines of treatment received by the patient before the biopsy. This result is in line with clinical data showing that overall tumor drugs sensitivities decrease along the course of the disease [44]. With time, the pilot PDO cohort may be enriched with new PDOs and drug tests to report the heterogeneity of the disease and refine hits identifications.

In this study, we could not orientate patient treatment based on the chemogram. Even so, we were able to monitor eight of the patients who received SOC treatment after we performed the chemogram on their PDOs. Various studies have investigated the predictive value of using PDOs to select optimal CRC treatment at different stages of disease in patients undergoing systemic chemotherapy [19]. The use of PDOs correctly predicted drug response with irinotecan-based chemotherapy in patients with mCRC in the TUMOROID study [36] and in patients with locally advanced rectal cancer in the ClinCare study [30]. However, for oxaliplatin-based therapy, results are more mixed: Ganesh et al*.* found satisfactory results for rectal cancer patients [45] while in the TUMOROID [36] and in APPOLLO [35] studies, drug screen results for mCRC patients were not satisfactory. In this study, among the eight patients we could follow, six of them showed clinical concordance with the chemogram. Although the number of evaluable patients is relatively small, we did not observe any bias for oxaliplatin-based regimens in our cohort.

The full implementation of ex vivo drug tests for functional precision medicine strategies will require to tackle remaining challenges. These include testing combination therapies, assessing the contribution of intra-tumoral heterogeneity and incorporating the tumor microenvironment to test for immune-oncology therapies. Yet, along with other studies, our work proved that the PDO technology is mature to be tested in clinic to identify unexpected therapeutic options for refractory patients. We also identified clear parameters to improve the standardization and throughput of the ex vivo drug test that will likely improve the implementation of FPM. As a follow-up study, we opened the prospective multicenter ORGANOTREAT clinical trial (NCT05267912). The first segment, ORGANOTREAT-01 is a phase I/II study evaluating the feasibility and efficacy of PDO-based precision medicine in patients with refractory metastatic CRC. Together with other trials, this study will contribute to determine the patients who will benefit from functional precision oncology.

### Supplementary Information

**Additional file 1: Supplementary Table 1. **Supplementary clinical data. **Supplementary Table 2**. Annotations Fig. 2D. **Supplementary Table 3.** Supplementary clinical concordance data.

**Additional file 2: Supplementary Fig. 1.** (A) Test of 3 tumor digestion protocols based on the evaluation of viable cell number and cell viability signal. (B) CK20 and CDX2 staining. Scale Bar, 300 μm. (C) Bar graph of the total number of COSMIC referenced mutations per PDO. Data are presented as the mean + SD in A (*n* = 3). Significance is determined with one‐way ANOVA with Tukey’s multiple comparisons test in A. **Supplementary Fig. 2.** (A) Graph presenting PDO area as a function of relative ATP signal for 3 different PDOs: CGR002, CGR0029, CGR0039. Each dot is the average area of > 50 PDOs and the relative ATP signal of the entire well. 8 dots are displayed, representing 8 different conditions: 2 drugs (5FU and Oxaliplatin) at three different concentrations and one control condition per drug. (B) Graph A is annotated with 3 representative images of CGR0002 single PDO outlined with a pink line delimiting the segmentation zone for area extraction. (C) Graph presenting PDO area as a function of relative ATP signal for CGR0002 tested with 25 drugs at 3 concentrations in triplicate. Each dot is one well. (D) Graph presenting relative LDH signal as a function of relative ATP signal for CGR0002 tested for 6 drugs (5FU, Azacitidine, Bortezomib, Carboplatin, Cobimetinib and Docetaxel) at 3 concentrations in triplicate. Each dot is one well. Correlation is determined with the Pearson correlation coefficient (R) in A, C and D. **Supplementary Fig. 3.** (A) Drug test technical reproducibility for the other replicate combinations (supplementary data of Fig. 3E) assessed by the viability percentage obtained over one experimental run. (B) Bar graphs of the response of CGR0002, CGR0029 and CGR0039 to 5FU and Oxaliplatin before and after the freezing/thawing process. (C) Drug test biological reproducibility (supplementary data of Fig. 3F). Data are presented as the mean of triplicates in A (CGR0025 screened four times on the same run with 4 drugs at 3 concentrations (12 dots)), as the mean + SD of technical triplicates in B, as the mean of triplicates in C (CGR0002 screened on two different runs with a 2-week interval for 25 drugs at 3 concentrations (75 dots). Correlation is determined with the Pearson correlation coefficient (R). **Supplementary Fig. 4.** Sensitivity of the 25 PDOs to 24 drugs at 3 concentrations (supplementary data of Fig. 3G, 1 color line/PDO). Data are presented on the right panels as the mean response ± SD of the 25 PDOs to each drug. **Supplementary Fig. 5.** (A) Scatterplot of the PDO scores to 4 different couples of drugs (supplementary data of Fig. 4B). Each dot represents the score of one PDO. (B) Heatmap of scores for all 25 drugs against the 25 PDOs. Drugs were clustered according to their belonging to the CRC therapeutic arsenal (CRC SOC). Correlation is determined with the Pearson correlation coefficient (R) in A.

## Data Availability

The datasets generated and analyzed during the current study are available from the corresponding author on reasonable request.
