# Supplementary material for: Implementing patient derived organoids in functional precision medicine for patients with advanced colorectal cancer
Source: J Exp Clin Cancer Res. 2023 Oct 25;42:281. doi: 10.1186/s13046-023-02853-4 (PMC10598932; doi:10.1186/s13046-023-02853-4)

Supplementary Figure 1

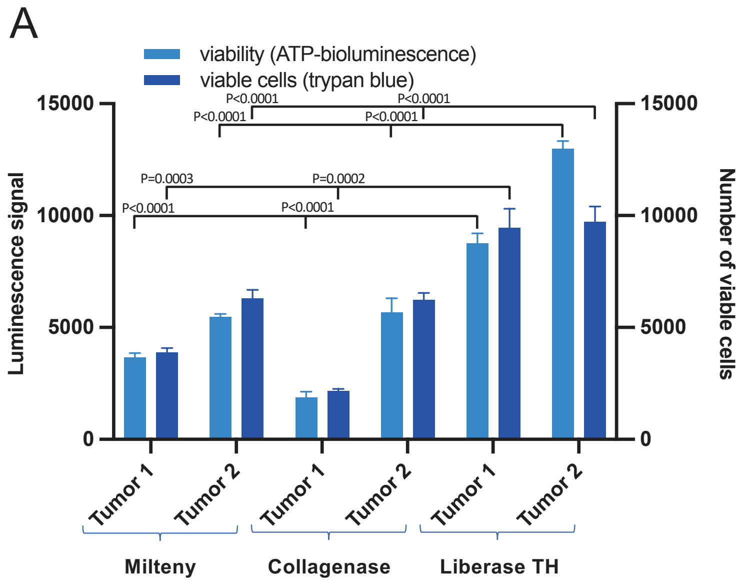

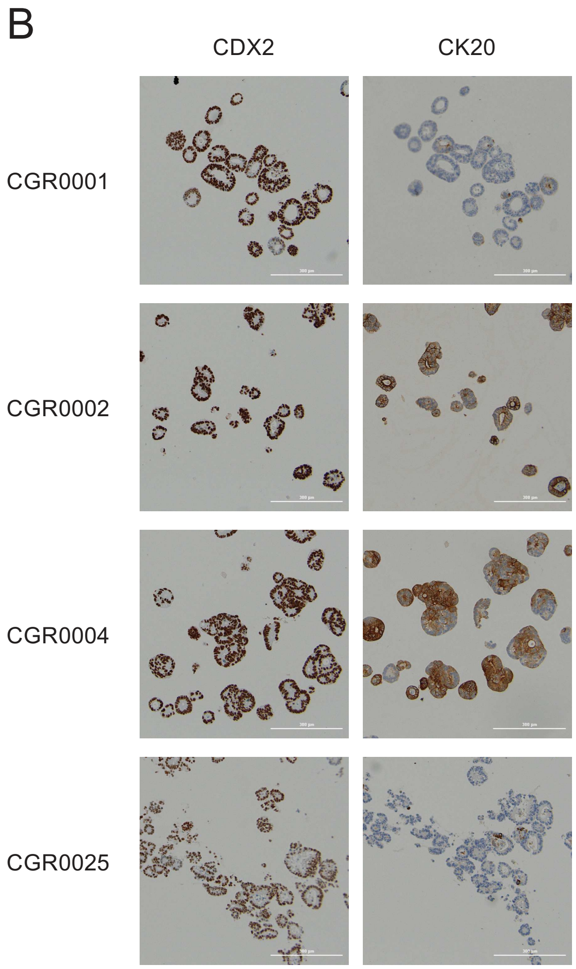

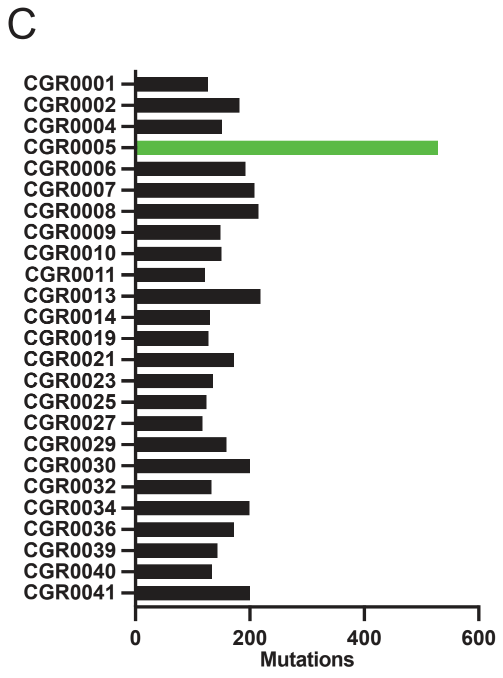

Supplementary Figure 2

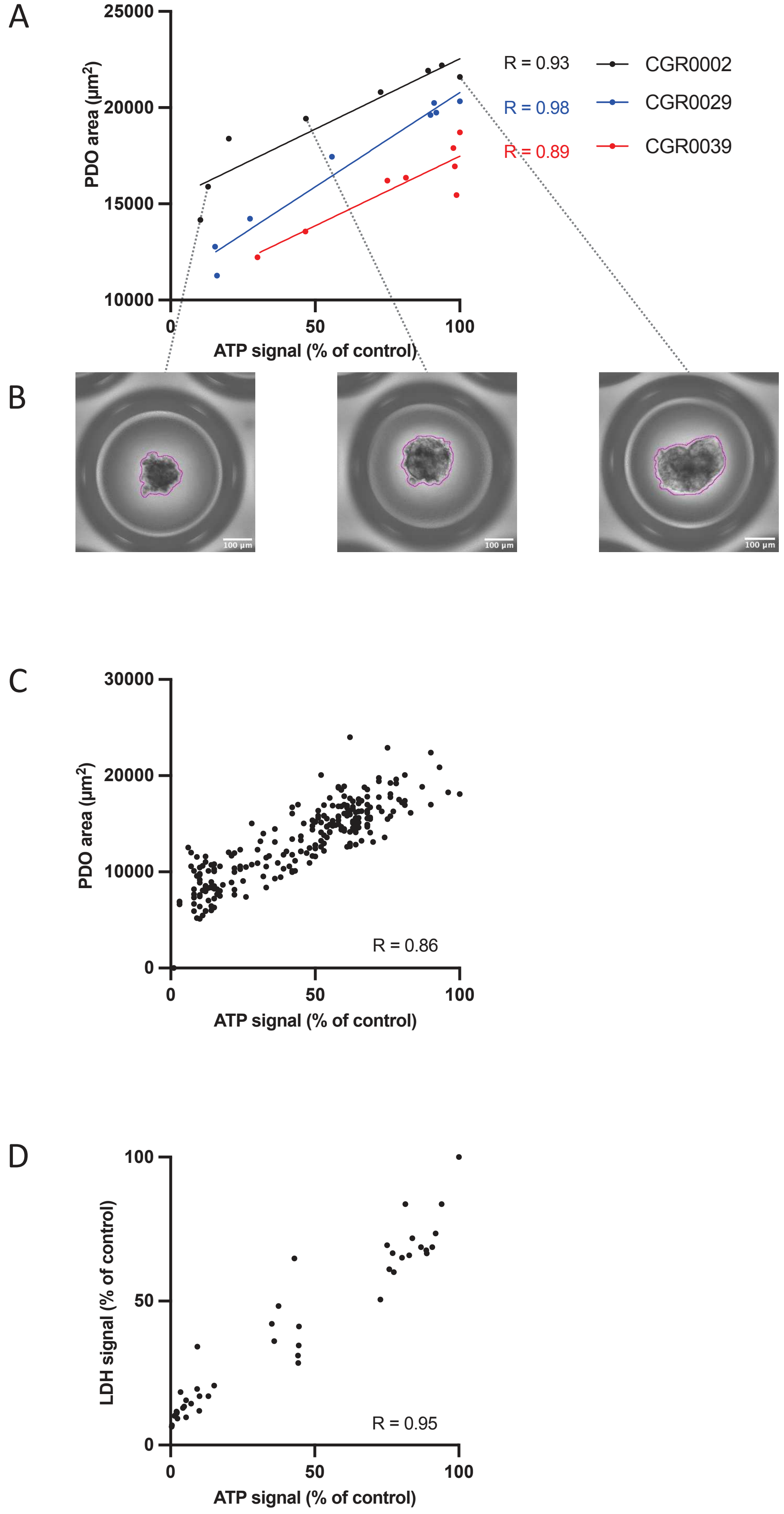

Supplementary Figure 3

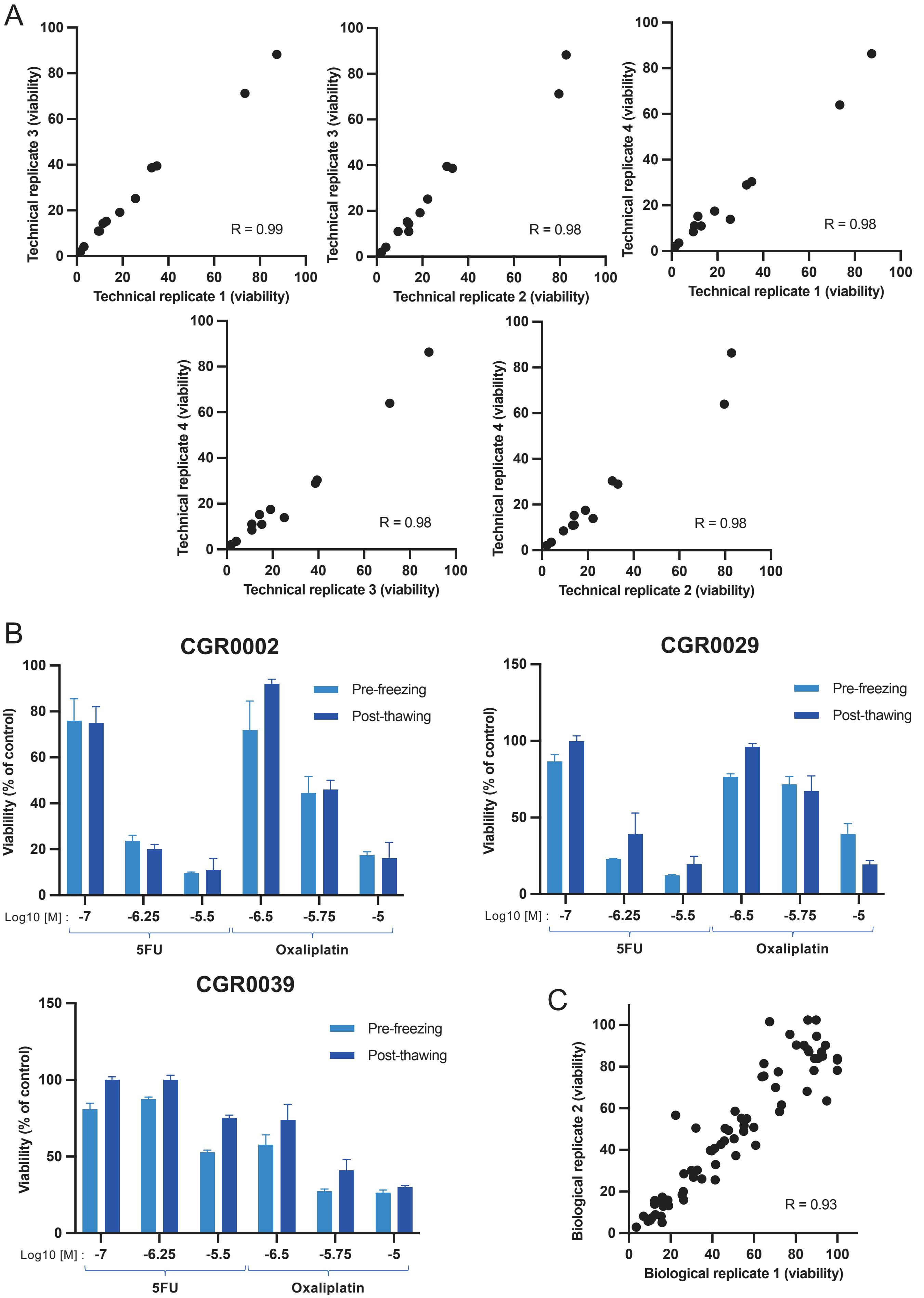

Supplementary Figure 4

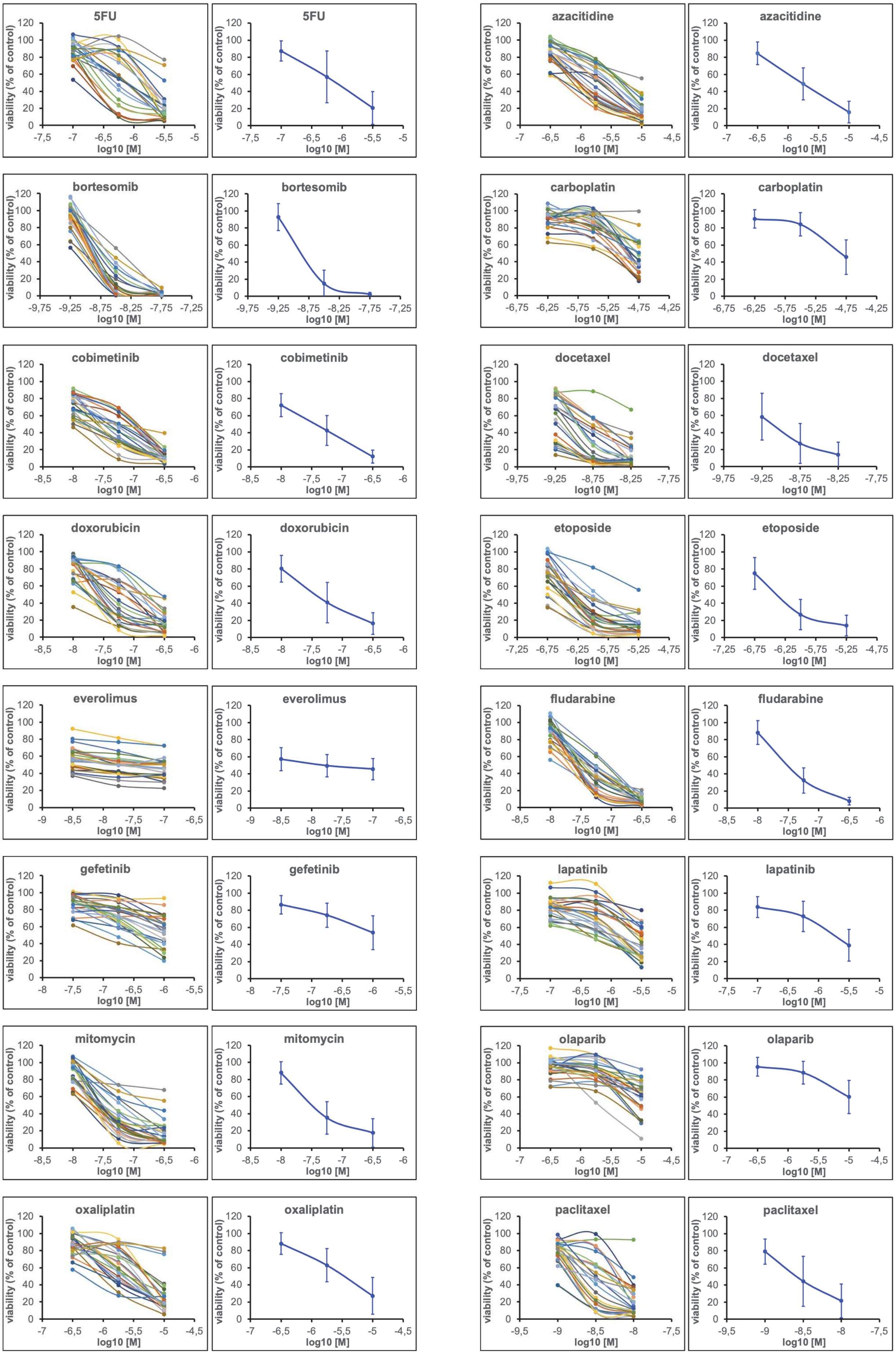

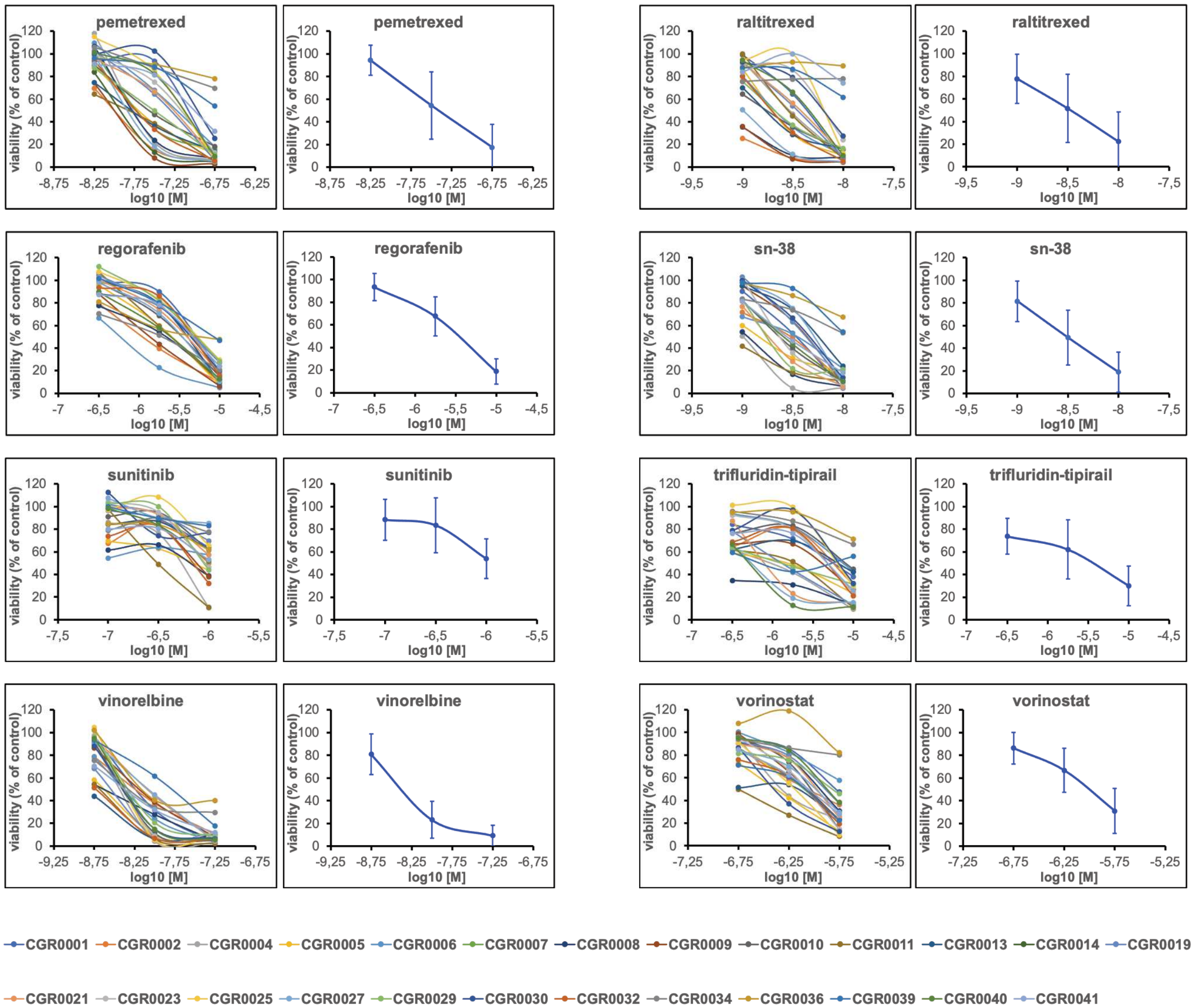

pemetrexed
viability (% of control)
log10 [M]
-8,75 -8,25 -7,75 -7,25 -6,75 -6,25
raltitrexed
-9,5 -9 -8,5 -8 -7,5
regorafenib
-7 -6,5 -6 -5,5 -5 -4,5
sn-38
sunitinib
-7,5 -7 -6,5 -6 -5,5
trifluridin-tipirail
vinorelbine
-9,25 -8,75 -8,25 -7,75 -7,25 -6,75
vorinostat
-7,25 -6,75 -6,25 -5,75 -5,25
0 20 40 60 80 100 120
CGR0001
CGR0002
CGR0004
CGR0005
CGR0006
CGR0007
CGR0008
CGR0009
CGR0010
CGR0011
CGR0013
CGR0014
CGR0019
CGR0021
CGR0023
CGR0025
CGR0027
CGR0029
CGR0030
CGR0032
CGR0034
CGR0036
CGR0039
CGR0040
CGR0041

Supplementary Figure 5

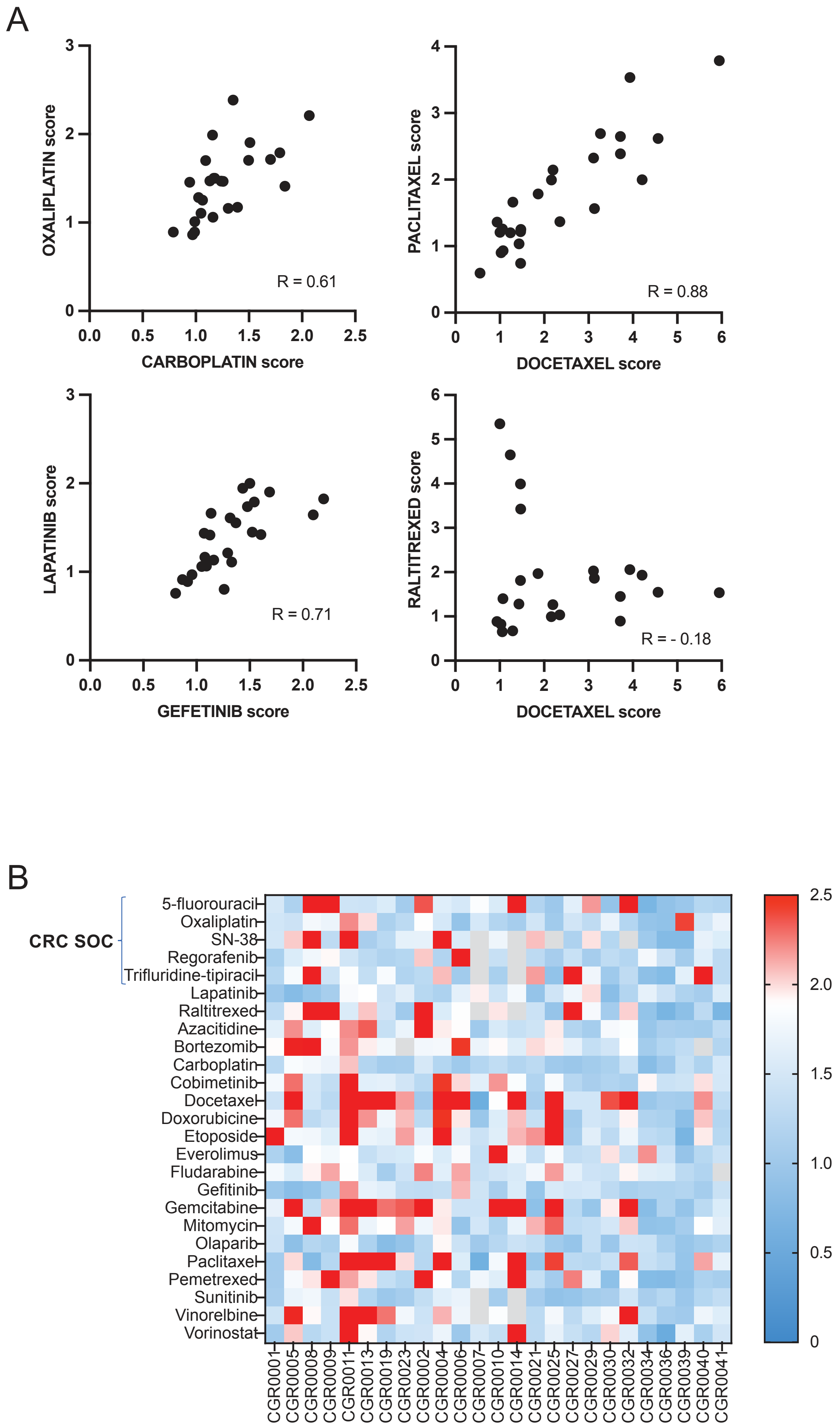

Supplement: Supplementary file 2 — Additional file 2: Supplementary Fig. 1. (A) Test of 3 tumor digestion protocols based on the evaluation of viable cell number and cell viability signal. (B) CK20 and CDX2 staining. Scale Bar, 300 μm. (C) Bar graph of the total number of COSMIC referenced mutations per PDO. Data are presented as the mean + SD in A (n = 3). Significance is determined with one‐way ANOVA with Tukey’s multiple comparisons test in A. Supplementary Fig. 2. (A) Graph presenting PDO area as a function of relative ATP signal for 3 different PDOs: CGR002, CGR0029, CGR0039. Each dot is the average area of > 50 PDOs and the relative ATP signal of the entire well. 8 dots are displayed, representing 8 different conditions: 2 drugs (5FU and Oxaliplatin) at three different concentrations and one control condition per drug. (B) Graph A is annotated with 3 representative images of CGR0002 single PDO outlined with a pink line delimiting the segmentation zone for area extraction. (C) Graph presenting PDO area as a function of relative ATP signal for CGR0002 tested with 25 drugs at 3 concentrations in triplicate. Each dot is one well. (D) Graph presenting relative LDH signal as a function of relative ATP signal for CGR0002 tested for 6 drugs (5FU, Azacitidine, Bortezomib, Carboplatin, Cobimetinib and Docetaxel) at 3 concentrations in triplicate. Each dot is one well. Correlation is determined with the Pearson correlation coefficient (R) in A, C and D. Supplementary Fig. 3. (A) Drug test technical reproducibility for the other replicate combinations (supplementary data of Fig. 3E) assessed by the viability percentage obtained over one experimental run. (B) Bar graphs of the response of CGR0002, CGR0029 and CGR0039 to 5FU and Oxaliplatin before and after the freezing/thawing process. (C) Drug test biological reproducibility (supplementary data of Fig. 3F). Data are presented as the mean of triplicates in A (CGR0025 screened four times on the same run with 4 drugs at 3 concentrations (12 dots)), as the mea [file 13046_2023_2853_MOESM2_ESM.pdf]
